# Low Temperature Plasma‐Assisted Double Anodic Dissolution: A New Approach for the Synthesis of GdFeO_3_ Perovskite Nanoparticles

**DOI:** 10.1002/smtd.202400481

**Published:** 2024-09-10

**Authors:** Natalie Tarasenka, Dilli Babu Padmanaban, Dmitry Karpinsky, Miryam Arredondo, Nikolai Tarasenko, Davide Mariotti

**Affiliations:** ^1^ Nanotechnology and Integrated Bio‐Engineering Centre (NIBEC) School of Engineering Ulster University Belfast Northern Ireland BT15 1ED UK; ^2^ Department of Design Manufacturing and Engineering Management University of Strathclyde Glasgow G1 1XJ UK; ^3^ Scientific‐Practical Materials Research Centre of NAS of Belarus Minsk 220072 Belarus; ^4^ School of Mathematics and Physics Queen's University Belfast Belfast Northern Ireland BT7 1NN UK; ^5^ B. I. Stepanov Institute of Physics National Academy of Sciences of Belarus 68‐2 Nezalezhnasti Ave. Minsk 220072 Belarus

**Keywords:** atmospheric pressure plasma, gadolinium orthoferrite, low‐temperature plasma electrolysis, magnetic nanomaterials, nanoparticles synthesis, perovskite oxides, plasma‐liquid interactions

## Abstract

Orthorhombic perovskite GdFeO_3_ nanostructures are promising materials with multiferroic properties. In this study, a new low‐temperature plasma‐assisted approach is developed via dual anodic dissolution of solid metallic precursors for the preparation of perovskite GdFeO_3_ nanoparticles (NPs) that can be collected both as colloids as well as deposited as a thin film on a substrate. Two solid metallic foils of Gd and Fe are used as precursors, adding to the simplicity and sustainability of the method. The formation of the orthorhombic perovskite GdFeO_3_ phase is supported by high‐resolution transmission electron microscopy, X‐ray diffraction, X‐ray photoelectron spectroscopy, and Raman measurements, while a uniform elemental distribution of Gd, Fe, and O is confirmed by energy dispersive X‐ray spectroscopy, proving the successful preparation of ternary compound NPs. The magnetic properties of the NPs show zero remnant magnetization typical of antiferromagnetic materials, and saturation at high fields that can be caused by spin interaction between Gd and Fe magnetic sublattices. The formation mechanism of ternary compound NPs in this novel plasma‐assisted method is also discussed. This method is also modified to demonstrate the direct one‐step deposition of thin films, opening up opportunities for their future applications in the fabrication of magnetic memory devices and gas sensors.

## Graphical abstract

A novel facile single‐step approach is introduced for the formation of perovskite nanoparticles in liquids based on atmospheric‐pressure low temperature plasma electrolysis in liquids. The precursor‐free method is capable of synthesis of gadolinium orthoferrite nanoparticles having perovskite orthorhombic structure showing as a prerequisite for a versatile plasma‐assisted synthesis of complex oxides nanomaterials.

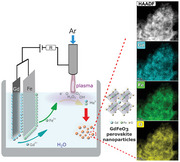

## 1 Introduction

Perovskite oxides nanoparticles (NPs) blend outstanding structure‐specific electronic, optical, and magnetic properties alongside chemical stability while offering materials sustainability. Perovskite oxides have a common ABO_3_ structure, where A and B are cations, being coordinated by 12 (site A) or by 6 oxygen atoms (site B). Stability and flexibility of the structure and variation of compositions occupying the perovskite structure substantiates the variety of properties demonstrated. As such, multiferroic properties, implying interdependent manifestation of ferromagnetism, ferroelectricity, and ferroelasticity, are of considerable interest for spintronics, electrically controlled magnetic memory devices, magnetic sensors, magneto‐optical materials, microelectromechanical systems (MEMS), catalysis, gas sensors, biomedicine, etc.^[^ ^1^, ^2^, ^3^, ^4^ ^]^ Owing to simultaneous ferroelectric and magnetic ordering, multiferroics allow to control charge and spin in the bound system by applying external magnetic or electric fields.^[^ ^5^ ^]^ This effect can be observed in GdFeO_3_ perovskite. GdFeO_3_ belongs to the orthoferrites family, demonstrating a combination of unique magnetic properties, such as room temperature antiferromagnetism, spin reorientations at low temperatures, spin switching,^[^ ^6^ ^]^ ferroelectricity,^[^ ^7^ ^]^ as well as distinctive magneto‐optical properties, including ultrafast photo‐magnetic excitation.^[^ ^6^, ^8^ ^]^ These properties stem from the existence of two magnetic orders within the material: antiferromagnetic from the Fe sublattice and paramagnetic from the Gd.

The physical characteristics of a multiferroic material are strongly sensitive to its lattice structure. The preparation of stoichiometric pure orthorhombic GdFeO_3_ compounds with distinct properties can be challenging because synthesis can result in the coexistence of cubic garnet (Gd_3_Fe_5_O_12_) and/or magnetite (Fe_3_O_4_) additional phases.^[^ ^9^ ^]^ The preparation of application‐ready orthorhombic GdFeO_3_ requires new synthetic phase‐selective procedures. At present, perovskite materials are prepared by mechanical grinding or solid state synthesis methods^[^ ^10^ ^]^ that require high temperatures. Alternatively, solution‐based chemical methods were recently developed and reported, among them sol–gel synthesis,^[^ ^11^ ^]^ co‐precipitation,^[^ ^12^ ^]^ sonochemical,^[^ ^13^ ^]^ hydrothermal synthesis,^[^ ^14^ ^]^ and heterobimetallic precursor methods.^[^ ^15^ ^]^ In general, perovskite materials are known to crystallize only at high temperatures (800–1200 °C). Therefore, the solution‐based methods require additional post‐synthesis annealing for the formation of crystalline structures. Synthesis at low temperatures is difficult thermodynamically and require kinetically driven mechanisms that allow overcoming the activation barrier of the perovskite phase formation. Overcoming this limitation requires innovative strategies to induce crystallization at more moderate temperatures, while still offering the flexibility needed to enhance and tune materials’ properties, particularly for the synthesis of advanced materials, including crystalline multi‐metal oxide thin films for microelectronics.^[^ ^16^ ^]^ Several approaches, typically based on wet and plasma chemistry and recently on the photochemical processes for inducing the crystallization at lower temperatures have been proposed.^[^ ^16^ ^]^ For example, sputtering relies on kinetic energy of the sputtered material to overcome the threshold activation energy value.

Nevertheless, to improve the performance of perovskite nanomaterials and to extend their application area, the development of novel synthesis approaches that can provide the control of the materials properties are required. Plasma generated at atmospheric pressure in or in contact with liquid can induce non‐equilibrium chemical processes not accessible during the conventional chemical or physical NPs production methods, that significantly expands the range of the nanomaterials produced in terms of their composition and structure. Plasma‐induced non‐equilibrium electrochemistry is a “young” field of research and to the best of authors’ knowledge this is the first attempt to synthesize complex multi‐element oxides by this method.

Here, a new one‐step low‐temperature approach based on simultaneous plasma‐induced dissolution of solid metallic precursors for the synthesis of perovskite oxide NPs is proposed. A distinctive feature of this method is represented by the supply of the required metallic components from simple solid metal foils and subsequent synthesis in an aqueous solution. The absence of any soluble chemical precursors or stabilizers simplifies the process to achieve higher purity products and contributes to the manufacturing sustainability. Furthermore, to meet specific application requirements, it has been possible to demonstrate direct formation of thin films, as an alternative to the production of NPs in colloids. This approach presents advantages for instance compared to existing methods.^[^ ^9^ ^]^ The resulting thin films are shown to be uniform, indicating promising prospects for this technique and the synthesis of perovskite NPs.

## 2 Plasma‐Induced Non‐Equilibrium Electrochemistry for the Synthesis of Colloidal GdFeO_3_ Nanoparticles

### 2.1 Synthesis and Materials Characterization

**Figure 1:**
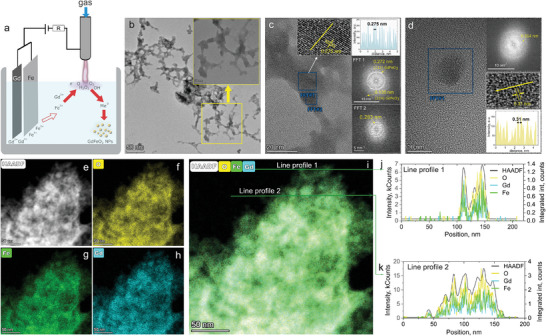
Synthesis of GdFeO_3_ perovskite NPs by plasma‐assisted dual anodic dissolution method: a) scheme of the experimental setup; b) TEM image of a selected group of NPs with the inset showing the agglomerated NPs at higher magnification, HRTEM images of the agglomerated nanostructures c) and separated spherical NPs d) with the insets showing the corresponding FFTs of the indicated regions. e) HAADF‐STEM image and corresponding EDX elemental maps of the group of particles for O f), Fe g), and Gd h); i) overlapped elemental map of the same group of NPs and j,k) are the line profiles along the directions indicated in Figure 1i.

The setup used in the experiments is depicted in **Figure** **1**a where an electrical discharge is generated and sustained over the surface of an aqueous solution. Such an approach allows production of NPs in colloid that is evidenced by the solution acquiring orange color within several minutes after the synthesis starts. The transmission electron microscopy (TEM) results confirm the successful preparation of crystalline orthorhombic non‐spherical GdFeO_3_ NPs that appear for the most part agglomerated into elongated branched structures (Figure 1b,c). This type of agglomeration could be attributed to their magnetic interactions.^[^ ^17^ ^]^ However, occasionally we could observe small isolated near‐spherical NPs as shown in Figure 1d. The elemental maps (Figure 1e–i), acquired by scanning TEM (STEM) energy‐dispersive X‐ray spectroscopy (EDX) indicate that the prepared NPs mainly consist of gadolinium, iron, and oxygen and that these are uniformly distributed across the agglomerates. This result is indicative of Gd, Fe, and O being bound in a compound or composite as further confirmed by high‐resolution TEM (HRTEM), X‐ray diffraction (XRD), Raman, and X‐ray photoelectron spectroscopy (XPS) results.

The HRTEM results disclose that agglomerated branched nanostructures consist of crystalline regions embedded into amorphous surroundings. The crystalline regions have crystal planes attributable to orthorhombic GdFeO_3_ as derived from the fast Fourier transform (FFT) processing of the HRTEM image. The inset in Figure 1c, corresponding to the region FFT1, shows lattice fringes with interplanar spacings of 0.272 and 0.135 nm, corresponding to the (112) and (224) planes of GdFeO_3_ of Pnma space group (JCPDS card number: 47–0067), expected at 2.72443 and 1.36221 Å, respectively. At a different region (FFT2), the interplanar spacing is found to be 0.283 nm that can be assigned to the (020) plane of orthorhombic GdFeO_3_.

The samples consisted for the most part of these GdFeO_3_ branched nanostructures, however, where we encountered isolated NPs (Figure 1d), with diameter <15 nm, these exhibited crystalline planes with spacings of 0.314 nm, corresponding to the (222) planes in cubic Gd_2_O_3_ (space group Ia $\bar{3}$). The presence of the separate gadolinium‐oxide particles is corroborated by SEM EDX results (see Figure 6; Figure S2, Supporting Information) that showed a slightly higher elemental concentration of Gd as compared to Fe. This excess Gd concentration can be attributed to the higher activity of Gd atoms as the standard electrode potential of Gd in the reaction Gd → Gd^3+^ + 3e^−^ (−2.279 V) is more negative than that of Fe in the process Fe → Fe^3+^ + 3e^−^ (−0.04 eV), indicating easier dissolution of the corresponding Gd atoms, leading to the crystallization in separate NPs.

**Figure 2:**
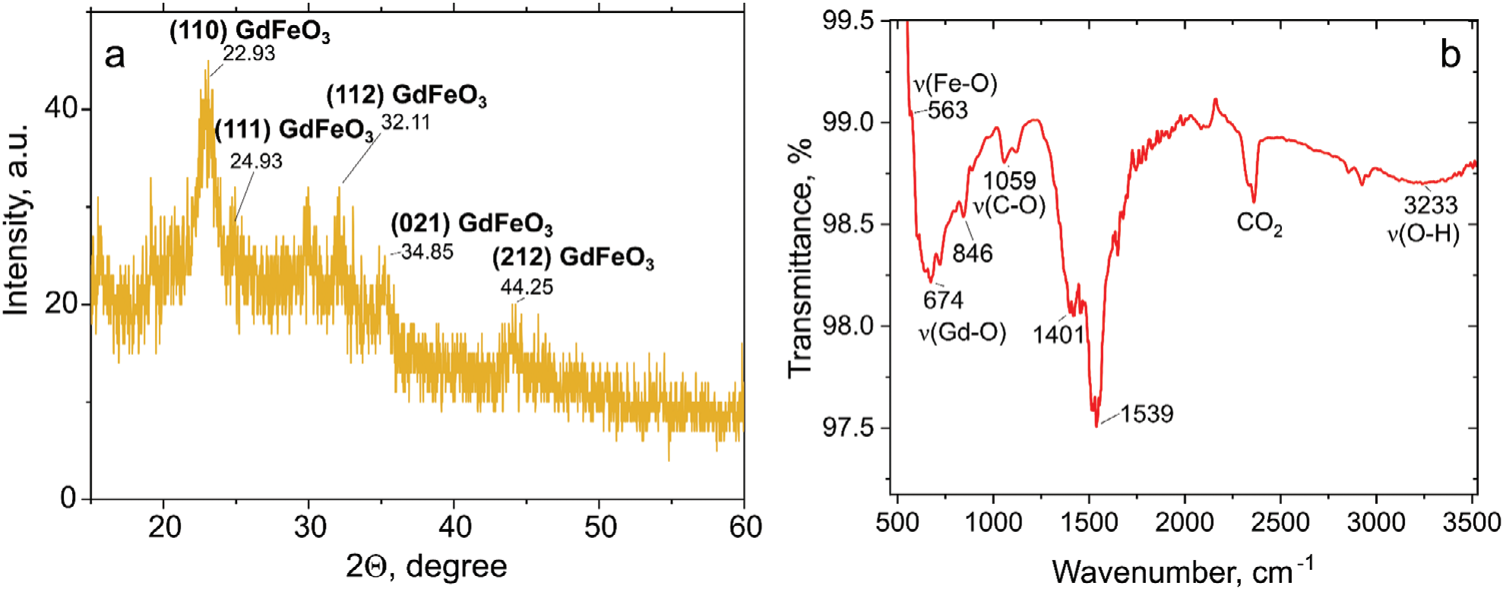
XRD pattern a) and FTIR spectrum b) of GdFeO_3_ NPs.

**Figure** **2**a shows a typical XRD pattern of the GdFeO_3_ sample. The broad and shallow characteristics of the peaks can be attributed to the small crystallites size as observed by TEM. The most prominent peaks are observed at 22.93°, 24.93°, 32.11°, 34.85°, and 44.25°, corresponding to the (110), (111), (112), (021), and (212) planes in accordance with orthorhombic GdFeO_3_ (JCPDS card number: 47–0067), found at 22.962°, 25.757°, 32.839°, 33.995°, 44.300°, respectively. XRD results confirm the formation of the orthorhombic perovskite crystalline GdFeO_3_ phase, in agreement with HRTEM results. The lattice parameters, calculated from the positions of the (110), (112) and (021) peaks using the equation 1/*d*_hkl_^2^ = *h* ^2^/*a* ^2^ + *k* ^2^/*b* ^2^ + *l* ^2^/*c* ^2^ (where d_hkl_ is the interplanar spacing), known for the materials having orthorhombic lattice, are as follows *a* = 5.509 Å, *b* = 5.689 Å, and *c* = 8.528 Å. These values are close to those typically reported for the orthorhombic phase of GdFeO_3_ (*a* = 5.349 Å, *b* = 5.610 Å, and *c* = 7.677 Å (JCPDS card number: 47–0067) with a lattice distortion, possibly caused by the size of the crystallites.^[^ ^18^, ^19^, ^20^ ^]^ The size of crystallites (*D*) was also determined from the XRD results using the Scherrer equation *D* = *Kλ*/*β*cos*θ*, where *K* represents the Scherrer constant (0.94), *λ* denotes the X‐ray wavelength (0.154 nm for Cu K_α_), *θ* is the diffraction angle, *β* is a full width at half maximum (FWHM) of the peak. Approximating the most distinct peaks in the XRD pattern (Figure 2) by Gauss profiling and taking into account the instrumental broadening (0.08°), resulted in an average value of 6.9 nm ± 0.2 nm for the crystallites size (see Section SI‐G, Supporting Information). This estimate is consistent with the particle sizes observed by TEM with sizes ranging from ≈5 to ≈8 nm (Figure 1b,c and see also Section SI‐G, Supporting Information).

Fourier Transform Infrared Spectroscopy (FTIR) analysis further confirms the composition of the NPs samples. In the FTIR spectra, the peaks related to the metal‐oxygen vibrations are usually observed in the range of wavenumbers 200–1000 cm^−1^ (Figure 2b). The broad peak in the range 560–670 cm^−1^ corresponds well to the literature data for the stretching vibrations of Fe─O and Gd─O bonds in Fe─O─Fe and Gd─O─Fe systems,^[^ ^21^, ^22^, ^23^ ^]^ which may overlap. The peak at 846 cm^‐1^ can be assigned to out‐of‐plane bending of the surface HCO_3_^−^ and CO_3_^2−^ groups, coordinated by Fe^3+^ or Gd^3+^ ions.^[^ ^4^ ^]^ As discussed in several works,^[^ ^4^, ^24^ ^]^ Fe and Gd ions in related oxides have a tendency to adsorb CO_2_ from the ambient air. This observation is also proved by the appearance of the bands corresponding to the mono‐ and bidentate carboxylic groups from adsorbed atmospheric carbon dioxide with vibrations at 1059, 1401, and 1539 cm^−1^ (see also Figure S9, Supporting Information). The adsorption of carbonate at the surface of metal oxides with subsequent metal ions coordination results in the symmetry disruption and activation of the IR vibrations due to the symmetry decrease. In addition, weak features attributable to the adsorbed water (O─H stretching vibrations at ≈3233 cm^−1^) were also observed in the FTIR spectra.

**Figure 3:**
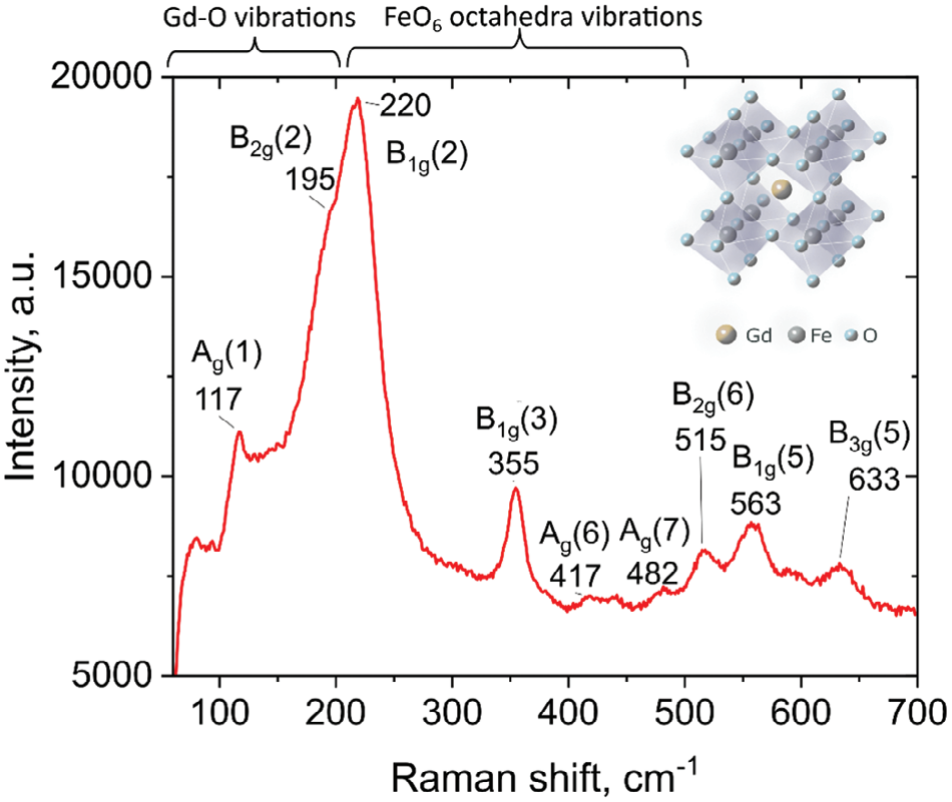
Raman spectrum of the GdFeO_3_ NPs.

Additional information about the crystal and defect structure of the NPs was obtained from Raman spectroscopy performed at room temperature in the range 100–1000 cm^−1^, shown in **Figure** **3**. The main sharp features appear at 117, 195, 220, 355, 515, 563, and 633 cm^−1^ accompanied by smaller peaks at ≈417 and 482 cm^−1^. The peaks appear to be symmetric, and their positions are in agreement with literature data for orthorhombic GdFeO_3_, known to have the most distinctive first order Raman peaks in the range of 100–650 cm^−1^ at room temperature.^[^ ^25^, ^26^, ^27^, ^28^, ^29^ ^]^ Typically, the Raman spectra of perovskite ferrites having orthorhombic structure of Pnma space group (D_2h_, 16) are characterized by 24 phonon modes, having the following distribution at the center of Brillouin zone: *Г*= 7*A_g_* +5*B*_1*g*_+7*B*_2*g*_+5*B*_3*g*._^[^ ^18^, ^26^ ^]^ Among these modes, those having A_g_ symmetry usually have the highest intensity. The vibration modes associated with the displacement of rare earth ions are located below 200 cm^−1^, while the phonon modes in the range of 200–400 cm^−1^ arise from different sources, including in‐ and out‐of‐phase FeO_6_ rotations and oxygen octahedral tilt.^[^ ^26^ ^]^ The modes within 450–500 cm^−1^ are associated with the oxygen octahedral bending vibrations in the O(1)─Fe─O(2) groups as well as with the modes arising from the stretching of Fe─O(1) and/or Fe─O(2) bonds. The mode at 633 cm^−1^ is related to in‐phase stretching or breathing vibration modes of FeO_6_ octahedra (B_3g_ (5)), which is normally found ≈630 cm^−1^.^[^ ^26^, ^27^, ^28^ ^]^ The latter peak is usually the most sensitive to the distortion of the lattice and formation of defects and therefore the shift of this peak may originate from distortion or defects from the lattice strain due to the small size and large surface to volume ratio of the nanoparticles. This is also corroborated by the broadening of the XRD peaks and typical of non‐equilibrium processes where defect formation and distortion are regularly observed.^[^ ^30^, ^31^, ^32^, ^33^ ^]^

The survey spectrum from XPS analysis also confirmed the elemental composition of the NPs, showing the prevalence of the lines attributable to Gd, Fe, and O with the presence of admixture of carbon (Figures S8,S9, and S11, Supporting Information). Further analysis of the high resolution XPS peaks allowed confirming Gd and Fe in the oxide structures having both +3 oxidation state as expected in GdFeO_3_.

**Figure 4:**
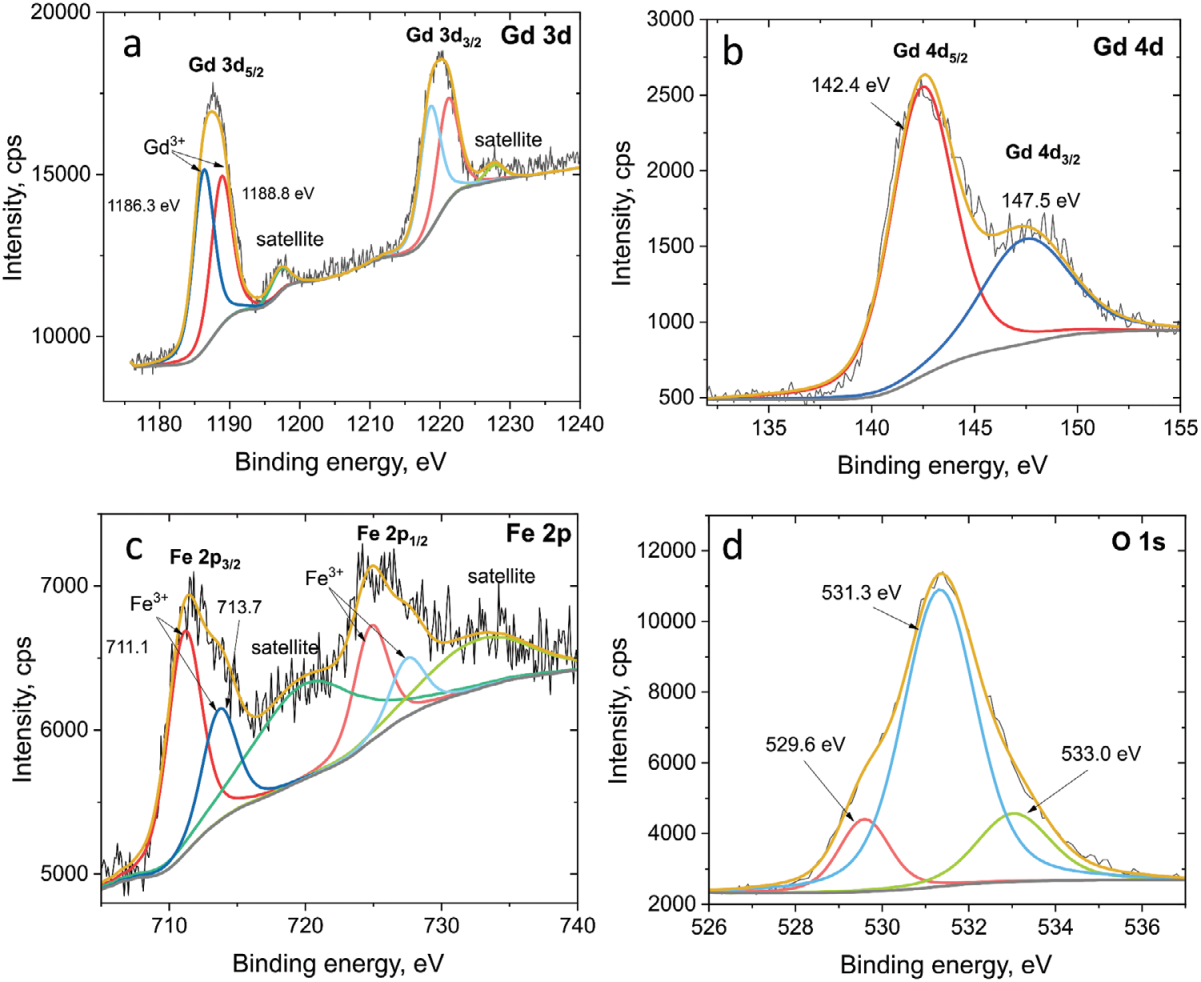
High‐resolution XPS spectra of a) Gd 3d, b) Gd 4d, c) Fe 2p, d) O 1s levels for the prepared GdFeO_3_ NPs.

In both Gd and Fe spectra, spin orbit coupling leads to the presence of doublets for p and d orbitals. Gd 3d peak tends to split into 3d_5/2_ and 3d_3/2_ (**Figure** **4**a). The deconvolution of the 3d_5/2_ signal allowed identifying the peaks centered at 1186.3 and 1188.8 eV, while the 3d_3/2_ was deconvoluted into the peaks at 1218.6 and 1221.1 eV. According to the position of these peaks, Gd is present in the sample as Gd^3+^. In addition, the presence of multiple peaks (satellites) in the 3d_5/2_ and 3d_3/2_ peaks of Gd was observed and is due to final state effects.^[^ ^34^ ^]^ The satellite peaks were located at ≈1197 and 1228 eV that is a typical feature of Gd^3+^ found in GdFeO_3_ nanostructures.^[^ ^35^, ^36^ ^]^

The analysis of the Gd 4d region (Figure 4b) additionally proves gadolinium appearing in the Gd^3+^ state. The Gd 4d core‐level spectrum also evidences a split as a result of the spin–orbit coupling occurring due to the 4d‐4f valence band electrons interaction.^[^ ^37^ ^]^ The peaks are here overlapping due to the small energy difference, which is proportional to the spin–orbit coupling constant and depends on the value 1/*r* ^3^ (*r* is radius) for the particular orbit.^[^ ^38^ ^]^ Satellite peaks appearing due to the final state effects are not resolved in the collected Gd 4d spectra, possibly because of the lower intensity of this peak compared to the Gd 3d one. The contributions center at ≈142.4 and 147.5 eV, are in agreement with the Gd 4d_5/2_ and Gd 4d_3/2_ values reported in the literature for the Gd^3+^.^[^ ^37^, ^39^, ^40^, ^41^ ^]^

The spectrum of Fe 2p also exhibits a spin‐orbit split into two components, 2p_3/2_ and 2p_1/2_, as it is typical of the iron‐based compounds.^[^ ^38^, ^42^ ^]^ The Fe 2p spectrum was deconvoluted into the peaks at 711.1 and 713.7 eV for the Fe 2p_3/2_ contribution. These positions correspond well to the Fe^3+^ ions occupying octahedral and tetrahedral positions in the GdFeO_3_ perovskite structure.^[^ ^18^, ^43^, ^44^ ^]^ No contribution from the peaks related to the Fe^2+^ oxidation states were observed in the spectra (expected at ≈709 eV),^[^ ^45^ ^]^ indicating that Fe appears only in the +3 state in these samples. The position of the satellite peak at ≈719 eV is also in agreement with the literature for Fe^3+[^ ^18^ ^]^ and its emergence is also due to final state effects. In case of iron, the appearance of the shake‐up satellite peak can be related to the 3d‐4s electron transition as a result of the core 2p photoelectron ejection.^[^ ^46^, ^47^ ^]^ As shown in several papers,^[^ ^38^, ^48^ ^]^ the satellite peaks position is very sensitive to the chemical oxidation states of iron: for Fe^3+^ it is observed ≈8 eV higher than the position of the Fe 2p_3/2_ peak while for the Fe in the +2 state this difference is lowered to ≈5–6 eV. The satellite for the Fe 2p_1/2_ peak appears at ≈732.8 eV.^[^ ^38^ ^]^

The analysis of the high‐resolution O 1s spectrum allows further understanding of the oxygen species present in the NPs. Figure 4d shows that the O 1s spectrum can be deconvoluted into several peaks at ≈529.6, 531.3, and 533.0 eV. The first contribution corresponds to the lattice oxygen anions appearing in oxide structures due to Fe─O and Gd─O bonds present in GdFeO_3_.^[^ ^18^, ^44^, ^49^ ^]^ Namely, the position of the oxygen peak ≈530 eV was associated with O^2−^ bonded to Gd in the GdFeO_3_ orthoferrite structure.^[^ ^44^ ^]^ The peak at 533.0 eV can be related to weakly adsorbed species such as water moisture from the surrounding atmosphere.^[^ ^43^, ^50^ ^]^

The deconvoluted peak ≈531.3 eV is dominant in the spectra and typically is related to the binding energy of surface oxygen states and surface bonded species, such as OH, O^−^, and O_2_^2‐^ that can be related to the distortion and oxygen deficiencies at the surface of perovskites.^[^ ^18^, ^49^, ^50^, ^51^, ^52^, ^53^ ^]^ For instance, Dupin et al.^[^ ^50^ ^]^ assigned the contribution in the range of 531–532 eV to the presence of O^‐^ ions on the surface due to partial coordination at the surface. This contribution can be quite significant in the O 1s peak and dominate over the lattice oxygen as previously observed also in Gd_2_O_3_.^[^ ^52^ ^]^ In our spectra, it is also possible that adsorbed carbonate species, as confirmed by FTIR and XPS, have also contributed further to increase the intensity at 531–532 eV.^[^ ^53^ ^]^ The presence of carbonates is also confirmed by the observation of the component at 289 eV in the C 1s spectrum (Figure S9) that is in agreement with FTIR results. Overall, the de‐convolution of the O 1s peak is consistent with the literature for perovskite oxides where several factors may contribute to different components simultaneously.

### 2.2 Magnetic Properties

**Figure 5:**
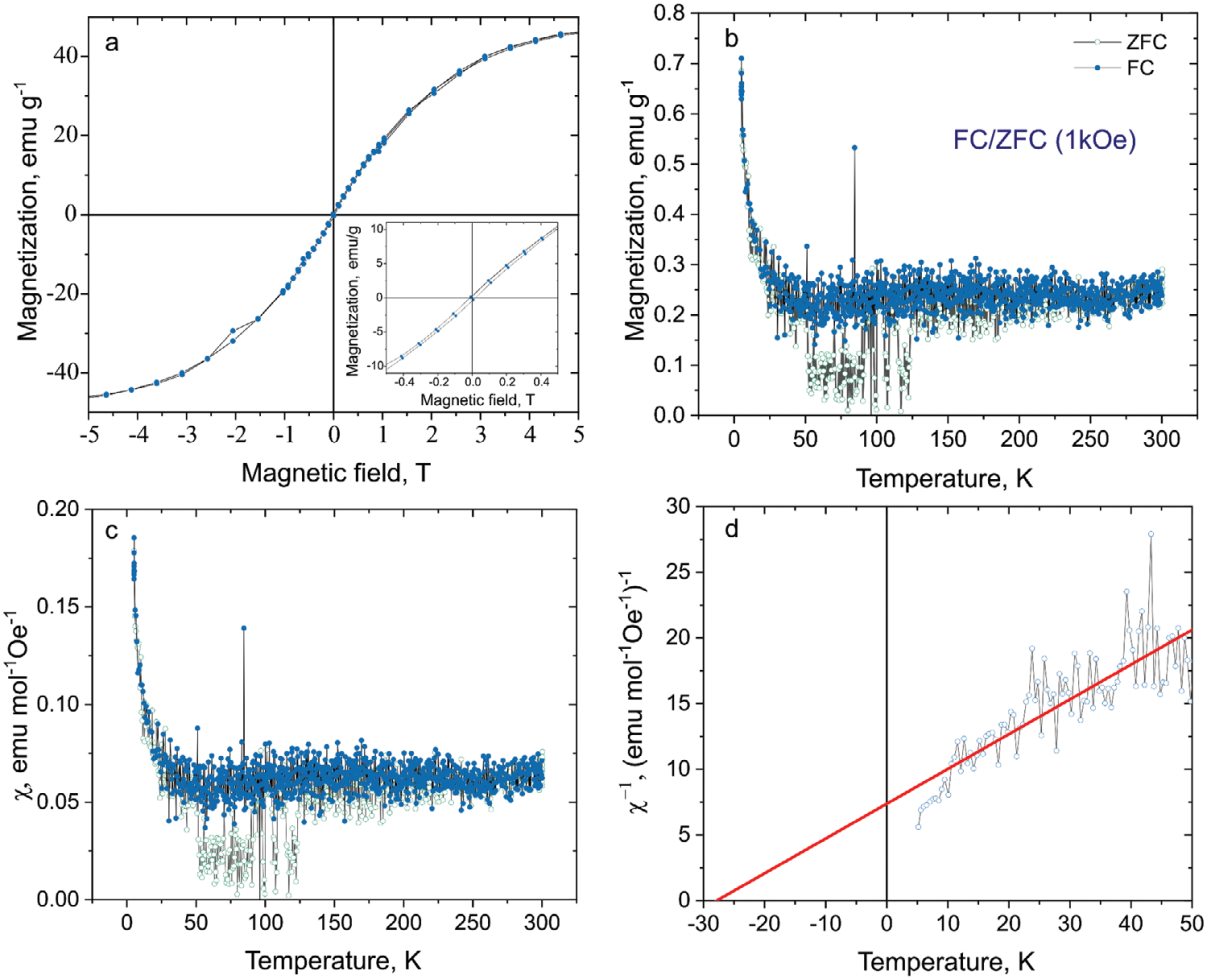
Isothermal magnetization M(H) curve traced for GdFeO_3_ at temperature 5 K a), the inset shows the magnified view of the observed small hysteresis near zero field region, and b) temperature dependence of the magnetization after ZFC and FC in GdFeO_3_ NPs measured at 1 kOe. Figure c) shows the dependence of magnetic susceptibility depending on temperature, d) – Curie‐Weiss (CW) fitting of ZFC χ^−1^ (T) plot.

The magnetization, relative to the applied field and temperature, was measured in the range ±14 Tesla at 5 K. The isothermal M(H) in **Figure** **5** shows zero remnant magnetization typical of antiferromagnetic materials. Saturation at high fields (∼45 emu/g) can be caused by the spin interaction between Gd and Fe sublattices as well as by the substrate contribution. Notably, this magnetic behavior further proves the NPs crystallization into the GdFeO_3_ structure, as the garnet Gd_3_Fe_5_O_12_ is known to exhibit ferrimagnetic properties.^[^ ^54^ ^]^

Typically, GdFeO_3_ exhibits canted antiferromagnetic behavior at room temperature that is attributed to its distorted perovskite structure with orthorhombic cell.^[^ ^6^ ^]^ Since the perovskite structure of GdFeO_3_ is formed by two sublattices, the magnetic properties, including the shape of the M(H) curve, are determined by three main contributions: antiferromagnetic input of the Fe oxide sublattice, paramagnetic contribution from the Gd ions^[^ ^55^ ^]^ as well as Gd^3+^‐Fe^3+^ exchange interactions.^[^ ^28^ ^]^ The antiferromagnetic contribution arises from the super‐exchange mechanism of spin coupling via Fe^3+^─O^2−^─Fe^3+^ groups. The paramagnetic contribution is attributed to the non‐coupled Gd^3+^ ions, that are known to have 7 unpaired electrons (half occupied 4f subshell). Both these contributions result in long range antiferromagnetic ordering of GdFeO_3_. When the particles size is reduced to the nanoscale, the dominant magnetic exchange interaction may change and influence the corresponding magnetic properties.

It is noteworthy that a very small hysteresis loop was also observed in the M(H) curves at 5 K (inset in Figure 5a), which is indicative of a weak ferromagnetism. This weak ferromagnetism is also typical for GdFeO_3_ and can be explained by the strain and distortion of the GdFeO_3_ structure due to the reduction of Gd^3+^ (A‐site cation) coordination number from 12 to 8^[^ ^56^ ^]^ or canted Fe sublattice.^[^ ^57^ ^]^ However, a weak ferromagnetism near zero field has been also attributed to NPs agglomeration,^[^ ^4^, ^12^ ^]^ which, together with the superparamagnetic behavior at 5 K, can be explained by alignment of the magnetic moments across different NPs. Still, the observed coercivity is very low and thus cannot significantly affect the overall magnetic behavior of the sample.

The contribution that defines the magnetic ordering of the material also depends on the temperature. GdFeO_3_ is characterized by a Néel temperature of 670 K, that corresponds to the transition from antiferromagnetic (G‐type) to paramagnetic characteristics. Below the Néel temperature, the antiferromagnetic contribution from the magnetic moments of the distorted FeO_6_ octahedra are dominant and this is usually explained by the strong exchange interaction in the Fe sublattice at high temperatures. As for Gd^3+^ ions, they are reported to be in the paramagnetic state in the temperature range ≈80–700 K.^[^ ^28^ ^]^ At very low temperatures around ≈10 K, the paramagnetic contribution of Gd^3+^ is expected to prevail. The interaction between Gd and Fe sublattices generally results in the appearance of the magnetic moment in Fe ions subsystem with Gd system remaining paramagnetic.^[^ ^6^, ^58^ ^]^ Several second‐order magnetic transitions are also known for GdFeO_3_ such as transitions from paramagnetic to weak ferromagnetic, spin‐reorientation transition of Fe^3+^ and antiferromagnetic ordering of Gd^3+^ below 2.5 K.^[^ ^28^ ^]^

To study the temperature dependence of the magnetization, measurements were performed using the same setup in the cooling regime at a constant field of 1 kOe (FC) and with no applied field (ZFC) (Figure 5b). The FC and ZFC measurements were used to determine the existence of superparamagnetic transitions and states in the NPs samples appearing isolated or in multi‐core environment.^[^ ^59^, ^60^ ^]^ For the ZFC measurements, the sample was first cooled down to the lowest temperature and the magnetization was measured while heating up the sample in an ≈1 kOe applied field. FC curves were obtained during cooling of the sample again in the same applied field. The results show that magnetization is absent until low temperatures are reached, in agreement with literature data for GdFeO_3_.^[^ ^6^, ^61^, ^62^ ^]^ Above 50 K, the ZFC and FC curves present different behaviors, which can be attributed to the induction of superparamagnetic transitions in the studied temperature range and to the formation of magnetic domains under the applied magnetic field.

The M(T) curves were used to plot the magnetic susceptibility (*χ* = M/H), see Figure 5c. Figure 5d presents the inverse of the magnetic susceptibility for a constant applied field of H = 1 kOe, determined from the ZFC curve. The data are then fitted in the temperature range of 0–50 K with a modified Curie‐Weiss (CW) law:^[^ ^63^ ^]^

(1) $$\chi \;=\chi_{0}\;+\frac{C}{T-\theta_{CW}}$$

where *χ_0_* is the temperature‐independent contribution to the susceptibility, *T* is the temperature, *C* is the Curie‐Weiss constant showing the slope and *θ_CW_* denotes the Curie‐Weiss temperature. The values for *θ*_CW_ and *C* can be determined by plotting the inverse of the susceptibility and fitting the linear region, where 1/*C* corresponds to the slope of the curve, the *x*‐intercept is the value for *θ*_CW_ and the *y*‐intercept is *θ*_CW_/*C*. Herein, the value we found for *θ*_CW_ was −27.8 K and *C* equals to 3.77. The negative value of the Curie‐Weiss temperature confirms the antiferromagnetic character of the GdFeO_3_ NPs.^[^ ^63^ ^]^ The Curie constant *C* is directly related to the number of unpaired electrons in the atoms. Using this value, the effective magnetic moment per ion can be calculated (in Bohr magnetons):

(2) $$\mu_{\mathrm{eff}}=\sqrt{8C}\;\mu_{B}$$

For our samples, the *µ*_eff_ was found to be 5.5 *µ*_B_ per formula unit.

To summarize, the NPs synthesized are expected to have antiferromagnetic behavior that is promising for a number of practical applications, including spintronics. Further opportunities are offered by the combination of antiferromagnetic ordering and ferroelectric properties.^[^ ^40^ ^]^ As shown by Tokunaga et al.,^[^ ^7^ ^]^ GdFeO_3_ crystals are characterized by ferroelectric polarization below the temperature of antiferromagnetic ordering of Gd^3+^, which originates from the confinement of the Gd^3+^‐Fe^3+^ exchange interaction.

### 2.3 Discussion of the Possible Mechanisms Leading to NPs Formation

In this work, we have focused mainly on demonstrating the feasibility of utilizing simultaneously two sacrificial solid electrodes to achieve double anodic dissolution and the synthesis of complex perovskite oxides. The synthesis parameter space was identified after optimization. While the synthesis parameter space is limited, exploring different synthesis conditions (e.g., varying discharge current, but also studying the impact of the geometry of the electrodes and experimental set‐up) may allow tailoring NP properties.^[^ ^64^ ^]^ For instance, the discharge current has been shown to impact the synthesis rate.^[^ ^65^, ^66^, ^67^ ^]^ However we should note that in the synthesis of metal oxides, as compared to the synthesis of metallic NPs, the balance between the supply of reduced metals and oxidizing agents is important.^[^ ^66^ ^]^ We should for instance expect that changes in the synthesis parameters may lead to the preferential formation of Gd‐oxides.

A crucial component impacting the synthesis is the solution composition. For instance, ethanol has been used to effectively control the size of NPs in simple metal oxides,^[^ ^66^, ^67^ ^]^ while water has shown to enhance the growth.^[^ ^68^ ^]^ The pH has also quite a significant effect.^[^ ^69^ ^]^ As we have optimized our synthesis process, we have experimentally observed that a pH higher than 7 prevents dissolution from the Fe foil. We therefore infer that the more acidic synthesis conditions are required to prevent or remove the oxide layer from the electrode and allow for dissolution to occur. This is corroborated by higher rates of anodic dissolution in acidic solutions as reported in the literature.^[^ ^65^, ^70^ ^]^ Radicals (e.g., ·OH) that may be involved in the oxidation mechanisms of the nanoparticles (see below^[^ ^71^ ^]^), are readily and intimately linked to the pH of the solution, hence, while representing a considerable challenge in the control and understanding of the synthesis mechanism, we should also expect many opportunities offered by exploring synthesis parameters leading to accurate manipulation of NP properties.

In our work, the synthesis of the GdFeO_3_ NPs is achieved at low‐temperature (<100 °C). The use of the plasma as an “electrode” provides access to different chemical pathways, not available through conventional electrolysis that uses a solid cathode immersed in the solution. Plasma‐induced electrochemistry supplies a variety of reactive species that can participate in the reactions with metal ions resulting from the metal electrode anodic dissolution. This method has been used to produce NPs from metals with an electrode potential higher than that of hydrogen, e.g. Au, Ag, and Cu.^[^ ^66^, ^72^, ^73^, ^74^, ^75^ ^]^ Also, NPs based on transition‐metals have been previously synthesized and for instance anodic dissolution of iron was used for doping TiO_2_ nanostructures,^[^ ^76^ ^]^ demonstrating that the anodic dissolution of iron was enhanced in acetylacetone/ethanol solutions and by acidifying the solution with the addition of iodine. In our work, acidification can occur due to dissolution of atmospheric carbon dioxide.^[^ ^77^ ^]^ The formation of oxide layers on the sacrificial metal electrode is also an issue that can prevent metal dissolution and NP formation. However, an appropriate composition of the solution can overcome this challenge. For example, plasma‐induced electrochemistry in an aqueous‐NaCl solution has been used to synthesize Ni(OH)_2_ nanosheets, showing the importance of Cl^−^ ions for the promotion of Ni^2+^ ions, preventing the formation of a stable nickel oxide layer.^[^ ^78^ ^]^ In our case, the synthesis was carried out in distilled water, having a pH of ≈5 due to the dissolution of CO_2_ that prevents the formation of the oxide layer on the Gd and Fe foils.

We therefore believe that the first step in the NP synthesis is represented by the dissolution of the metal anodes under the electric field, and specifically via one‐step three electron transfer reactions:^[^ ^66^ ^]^

(3) $$\mathrm{Gd}\to Gd^{3+}+3e^{-}$$

(4) $$\mathrm{Fe}\to Fe^{3+}+3e^{-}$$

Both diffusion and the presence of an electric field facilitates the migration of the ions toward the plasma‐liquid interface acting as the cathode.^[^ ^79^ ^]^

Electrons from the plasma, injected at the plasma‐liquid interface, can produce, directly or indirectly, a range of reactive species, such as solvated electrons (e_aq_ *^−^*), ·H, ·O, H_2_O_2_, ·OH etc.^[^ ^78^, ^80^, ^81^ ^]^ These are both strong reducing agents (e_aq_ *^−^*, H·) as well as oxidizing agents such as hydroxyl radical.

Among the reducing agents, hydrogen radicals are often considered to play a significant role in the next stage of the metal ions reduction process that can reduce the metal ions in several steps:^[^ ^81^ ^]^

(5) $$H\cdot +Me^{n+}\to Me^{\left(n-1\right)+}+H^{+}$$

where Me represents a metal, Gd and Fe in our case and *n* = 3, 2, or 1. Metal ion reduction however can also occur through electrons close to the plasma‐liquid interface:

(6) $$Me^{n+}+ne^{-}\to Me^{0}$$

As the hydrogen radicals as well as electrons have very short lifetime and shallow penetration depth into the liquid,^[^ ^82^, ^83^ ^]^ these processes occur close to the plasma‐liquid interface. Hence, this region is often considered to be the most active reaction zone of the process. Penetration of the plasma jet into the liquid creates a crater and a stagnation layer beneath the liquid surface, while the ions migrating there undergo the reduction/oxidation processes with further gradual coloration of the solution acquiring the orange color.

Further oxidation steps may take place both in the volume of the liquid close to the plasma‐liquid interface as well as in the bulk of the solution. The reactions occurring close to the interface are likely due to reactions between reduced metal atoms with ·OH.^[^ ^84^ ^]^ In our case, the oxidation mechanism occurring at the interface can be considered dominant, confirmed by the uniform distribution of the elements inside the NPs observed in the EDX maps (Figure 1) that is achievable if the reaction occurs in the same confined reaction zone. A second process, in the bulk of the solution, results from the reactions between metal cations and OH^−^ that may also result in metal oxide NPs production.^[^ ^78^, ^85^ ^]^ It can be assumed that the reactions through the second mechanism produce a minor admixture of single metal oxide phases found in the separate small spherical NPs in contrast to the interconnected aggregates comprised by GdFeO_3_.

Nucleation and growth are therefore believed to progress through van der Waals forces and Ostwald ripening.^[^ ^78^ ^]^ The NPs magnetic properties could have also played a role in the formation mechanisms, corroborated by a degree of agglomeration observed in our TEM results. As a possible mechanism, Bleier et al.^[^ ^86^ ^]^ described a reversible magnetic agglomeration approach, based on which the growth process is determined by the magnetic susceptibility of the growing particles. When this parameter reaches a critical value, the magnetic attraction can overcome repulsive forces.

## 3 Fabrication of Nanostructured GdFeO_3_ Thin Films

**Figure 6:**
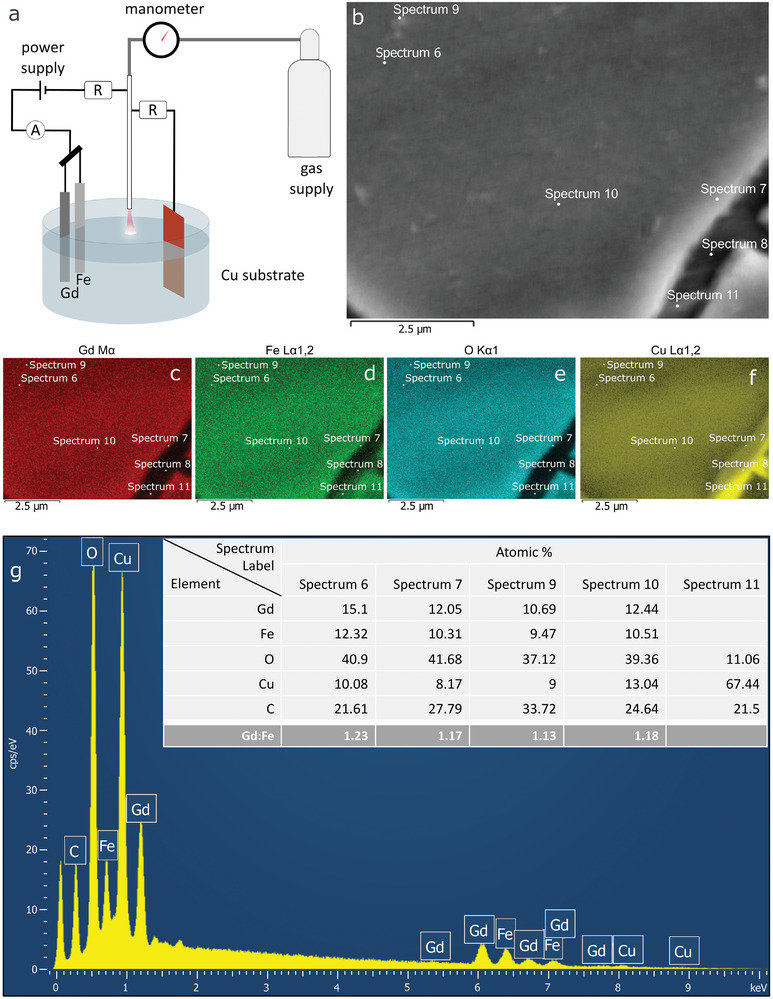
a) Scheme of the experimental setup used for perovskite NPs synthesis and simultaneous deposition, b) SEM image of the film deposited onto Cu substrate introduced in parallel to the plasma cathode; EDX elemental mapping of Gd Mα c), Fe Lα d), O Kα e) and Cu Lα f) in the selected area; Figure g) represents the EDX spectrum in the selected point, the inset table summarizes the composition (at.%) of the film in different points of the film.

To exemplify the versatility of this methodology, we have further explored the possibility of depositing the NPs directly on a substrate, forming NP thin films, and taking advantage of electrophoretic deposition of the colloid as NPs are formed.^[^ ^87^ ^]^ The method represents a simple and effective deposition approach, showcasing the flexibility and opportunities offered by plasma‐based electrochemical synthesis, applicable to a broad range of other conductive substrates (**Figure** **6**a). To enable the deposition, the conductive substrate was introduced in parallel to the plasma cathode through the ballast resistor. This allows attraction of the NPs to the substrate and their deposition into uniform thin‐films as shown in Figure 6b–g. SEM analysis shows a uniform film across tens of micrometers; along with some cracks attributable to the low wettability of the substrate, suggesting the necessity of an additional pre‐treatment prior to deposition.

EDX analysis indicates that the sample consists of Gd, Fe, and O (Figure 6c–g). The ratio of Gd and Fe atomic % varies in the range 1.13–1.23 that is close to the stoichiometric ratio for orthorhombic GdFeO_3_.

**Figure 7:**
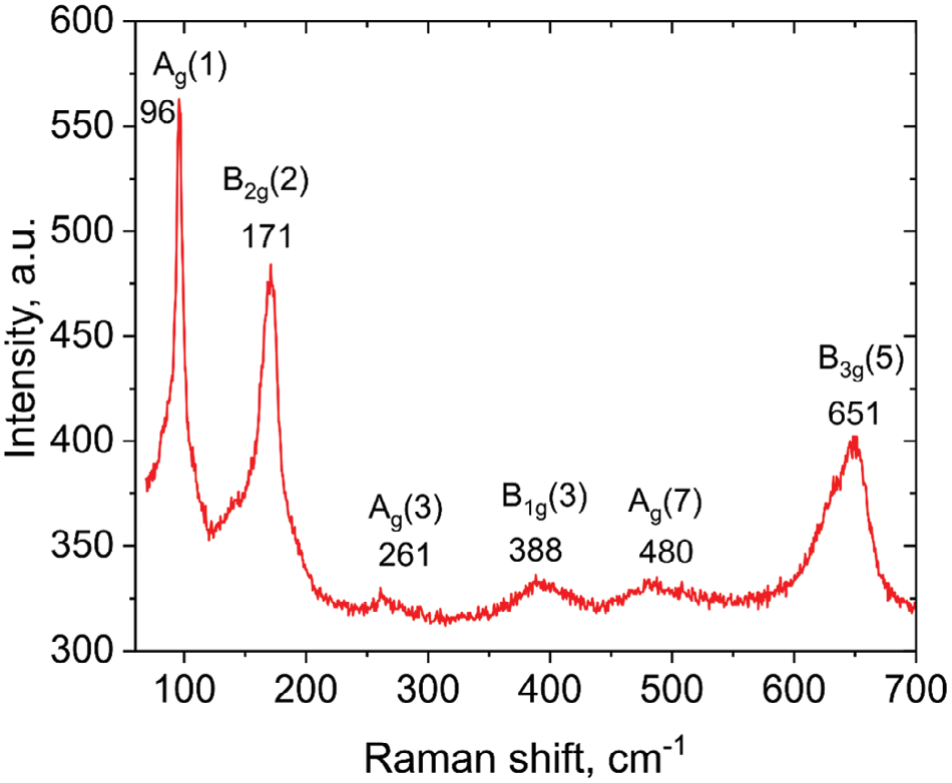
Raman spectrum of the GdFeO_3_ NPs deposited on the substrate during the synthesis process.

A typical Raman spectrum of the thin films is presented in **Figure** **7**. The results confirm that the deposited thin films have a similar structure to that of the NPs synthesized in colloid. Namely, the main peaks were found at 96, 171, and 651 cm^−1^ accompanied by smaller peaks at ≈261, 388, and 480 cm^−1^. The positions of the peaks are close to those observed for the NPs (Figure 3) in agreement with the literature data for the GdFeO_3_ structures. Thus, the developed approach allows synthesis and simultaneous deposition of the antiferromagnetic GdFeO_3_ nanostructured thin films. The successful demonstration of this technique opens a pathway for the novel versatile approach for complex multi‐metal oxide nanostructures fabrication.

## 4 Conclusion

In this work, a novel approach based on simultaneous plasma‐assisted anodic dissolution of constituent metals was proposed and tested toward the synthesis of gadolinium ferrite perovskite NPs and thin films. In the proposed synthesis scheme, metal foils of gadolinium and iron are used as solid precursors while NPs generation occurs as a result of the chemical reactions induced by the atmospheric pressure plasma at the interface between the capillary cathode and water surface. NPs characterization by HRTEM, SEM, EDX, XRD, FTIR, XPS, and Raman spectroscopy confirmed the formation of gadolinium ferrite having orthorhombic structure. EDX mapping shows uniform distribution of Gd, Fe, and O in the NPs and thin films while the Gd:Fe ratio was close to the stoichiometric value expected for GdFeO_3_. This approach also enables simultaneous synthesis and deposition of the NPs on a substrate.

The NPs exhibited a magnetic behavior consistent with GdFeO_3_ antiferromagnetic characteristics, with a zero remnant magnetization, a negative Curie‐Weiss temperature of −27.8 K and the absence of magnetization until low temperature is reached.

## 5 Experimental Section

### Experimental Details

The experimental setup is depicted in Figure 1a where an electrical discharge is generated and sustained over the surface of an aqueous solution (analytical‐grade double distilled water without any further purification). The discharge was powered by a stabilized direct‐current (DC) source (max. 15 mA, 10 kV, Matsusada Precision Inc.) with a voltage regulated up to a maximum of 1.5 kV to maintain a constant current of 4 mA (see Supporting Information for more details). As a cathode, a thin hollow stainless‐steel capillary was used with argon flowing through it at ≈60 standard cubic cm (sccm). The stainless‐steel cathode capillary (800 µm outer diameter and 500 µm inner diameter) was located at a distance of 2 mm above the water surface. As an anode, Gd and Fe foils (Alfa Aesar, purity 99.9%) were used and immersed in the aqueous solution at approximately a distance of 1.5 cm from the stainless‐steel capillary cathode.

A modified setup allowed simultaneous synthesis and electrodeposition of NPs to form thin films. In this case, an additional conductive substrate (copper foil, Fisher Scientific) was immersed in the 40 mL Pyrex glass beaker filled with distilled water. This substrate was connected to the power source in parallel to the plasma cathode through an additional ballast resistance (6.8 MΩ).

### Material Characterization

The composition, morphology, and optical properties of the resulting NPs and films were studied by X‐ray diffraction (XRD), X‐ray photoelectron spectroscopy (XPS), Raman spectroscopy, Fourier transform infrared spectroscopy (FTIR), scanning and transmission electron microscopy (SEM and TEM). To study the morphology, elemental composition, and crystalline structure, TEM and Scanning TEM (STEM) were performed using a Thermo‐Fisher Talos F200X G2 microscope operating at 200 kV, equipped with a FEG (field emission gun) and four in‐column Super‐X energy‐dispersive X‐ray (EDX) spectrometer detectors having a total collection angle of ≈0.9 sr., using a dwell time of 2.0 ms. For TEM studies, a 20 µL droplet of the prepared colloidal nanoparticles was drop‐casted on a holey‐carbon coated 200 mesh copper grid (Agar Scientific) and dried overnight at ambient conditions.

The morphology, structure, and elemental composition of the deposited nanostructures were also evaluated using field emission SEM (FE‐SEM) HITACHI SU5000 at accelerating voltage of 10 kV, equipped with a X‐MaxN 80 silicon drift detector for elemental analysis. To study the morphology of the NPs in the colloidal samples, a drop of the colloid was deposited onto Si substrate and dried at 80 °C. For SEM analysis of the thin films deposited onto Cu substrates during the synthesis, the sample was dried in air and attached to the microscope sample holder using a double‐sided copper tape.

Additional information on the phase composition, crystal, and defect structure of the prepared NPs was obtained from XRD, Raman, and FTIR analyses. For the XRD analysis, the colloidal sample with the NPs that remained in the reaction cell after synthesis, was drop‐casted onto a silicon substrate and dried at 80 °C on a hot plate and then analyzed by a Bruker D8‐Discover X‐Ray diffractometer with a Cu Kα radiation source. The XRD analysis was performed at room temperature in the 2θ range from 10° to 80°. The phase identification was performed by the comparison of the measured peaks with the JCPDS database entries.

For Raman spectroscopy, FTIR, and XPS, the colloidal samples were deposited onto an aluminum foil and dried at 80° C on a hot plate to remove excess water. FTIR spectra were collected using a Fourier spectrometer Thermo Fisher Nicolet iS5 (Termo Nicolet, USA) in the 4000–525 cm^−1^ range. Raman spectra were registered in the range of 50–3000 cm^−1^ using a scanning probe confocal microscope∖spectrometer Renishaw inVia Qontor Confocal Raman Microscope (Renishaw Ltd., UK). In the Raman studies, a 532 nm laser excitation source was used. The surface composition and valence states of Gd and Fe in the prepared NPs were determined by X‐ray photoelectron spectroscopy (XPS) using a ESCALAB XI+ spectrometer (Thermo Fisher, East Grinstead, United Kingdom) with Al Kα as the radiation source (excitation energy 1486.68 eV) having power 225 W (15 kV and 15 mA). The recorded high‐resolution spectra for C 1s, O 1s, Fe 2p, Gd 3d, and Gd 4d were collected with energy step of 0.1 eV, while for the survey spectra the energy step was 1 eV. The analyzed spot size was 650 µm. For charge compensation purposes, a flood gun was operated at 100 µA. The charge shift of the energy scale of the spectrometer was calibrated using shifting of the position of adventitious carbon peak to the 284.8 eV binding energy. The measurement of the magnetic properties was performed using Physical Properties Measurement System (Cryogenic Ltd., UK) in magnetic fields up to 14 Tesla in the temperature range of 5–300 K. For the magnetic measurements, the sample was prepared by drop‐casting of the synthesized colloidal sample onto a glass‐ceramic substrate and dried at 80 °C on a hot plate.

### Statistical Analysis

In this study, each synthesis experiment was tested three–five times, and each synthesized sample was characterized by TEM, SEM, EDX, XRD, XPS, Raman, and FTIR. For the determination the main parameters of the synthesized NPs, for example elemental composition by EDX, the results were presented as an average value and ± the standard deviation (Figure S2 (Supporting Information)). The measurement of the magnetic properties was performed using Physical Properties Measurement System and software of Cryogenic Ltd., UK. Statistical analysis was performed using OriginPro 2022 (Originlab). For analysis of the crystallite size from the XRD results, the Scherrer equation was used. An approximation of the most distinct peaks in XRD pattern by Gauss profile was used taking into account the instrumental line broadening (0.08°). The average value estimated by analysis of three most intensive peaks was found to be (6.9 ± 0.2) nm. ImageJ Fiji software was used to determine the interplanar spacings from the HRTEM results, the EDX mapping and line profile analysis as well as FFT processing of the high‐resolution TEM was done using Velox software (Thermo Fisher Scientific). The fitting of the XPS peaks has been performed using the Thermo Avantage software. For all the spectra, Shirley‐type background was used. The fitting was performed using mixed Gauss‐Lorentz curves (L/G 30%). The constraints for the peaks positions, widths, and areas were applied based on the literature data for the studied system. Among those, the FWHM of the 3d_5/2_ and 3d_3/2_ peaks of Gd and in the 2p_3/2_ and 2p_1/2_ of Fe were fixed and the ratio between the main peak and its spin orbit satellite were constrained to be ≈1:2 for the Fe 2p and 2:3 for Gd 3d.

## Conflict of Interest

The authors declare no conflict of interest.

## Supporting information



Supporting Information

## Data Availability

The data that support the findings of this study are available from the corresponding author upon reasonable request.
