# Supplementary material for: Low Temperature Plasma‐Assisted Double Anodic Dissolution: A New Approach for the Synthesis of GdFeO3 Perovskite Nanoparticles
Source: Small Methods. 2024 Sep 10;8(12):2400481. doi: 10.1002/smtd.202400481 (PMC11671862; doi:10.1002/smtd.202400481)
Supplement: Supplementary file 1 — Supporting Information [file SMTD-8-2400481-s001.docx]

Supporting Information

Low temperature plasma-assisted double anodic dissolution: a new approach for the synthesis of GdFeO_3_ perovskite nanoparticles

Natalie Tarasenka*, Dilli Babu Padmanaban, Dmitry Karpinsky, Miryam Arredondo, Nikolai Tarasenko, Davide Mariotti*

**SI-A. Electrical characterization of the plasma process**


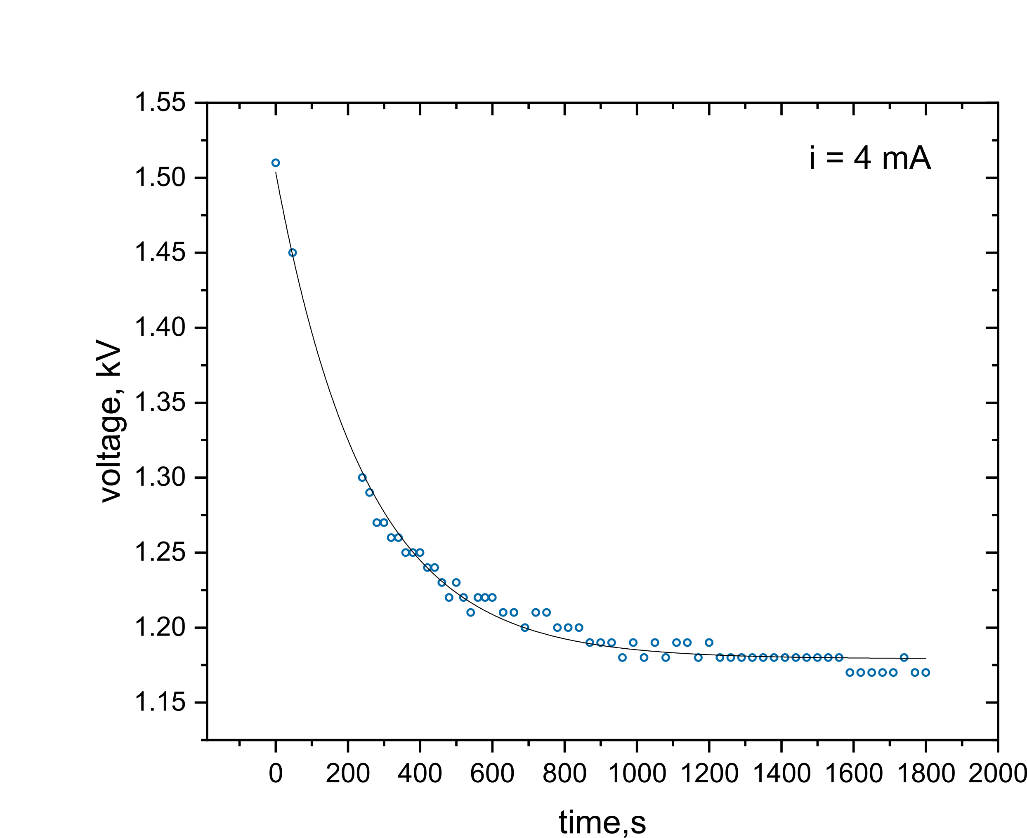


**Figure S1**. Change of the voltage with time from the start of the synthesis

**SI-B. Further SEM and TEM analysis of the NPs**





**Figure S2**. SEM analysis of the NPs prepared by dual anodic dissolution of Gd/Fe in distilled water and drop-casted from the colloid onto Si plate and dried at 80°C on a hot plate: a – EDX map showing uniform distribution of Gd, Fe and O in the formed nanomaterial, b – EDX spectrum of the area shown in figure S2a, c – SEM image of the group of nanoparticles, d-h – EDX maps of the Fe Lα (d), Gd Mα (e), O Kα (f), Si Kα (g) and C Kα (h), respectively. The atomic content of the Fe, Gd, O, Si and C was determined to be (10.5±0.1)%, (14.8±0.1)%, (46.8±0.3)%, (16.0±0.1)%, (12.0±0.2)%, respectively





**Figure S3**. NPs morphology and composition TEM (a) and STEM-EDX analysis (b-g) of the prepared GdFeO_3_ NPs; elemental maps of Gd (c), Fe (d) and O (e) revealing uniform distribution of the elements in NPs with an overlay in (b); (f) – EDX spectrum line profile from the area marked by an arrow in (b) as additional evidence of Gd and Fe simultaneous presence in NPs; (g) – EDX integrated spectrum of Area 1 in (b)


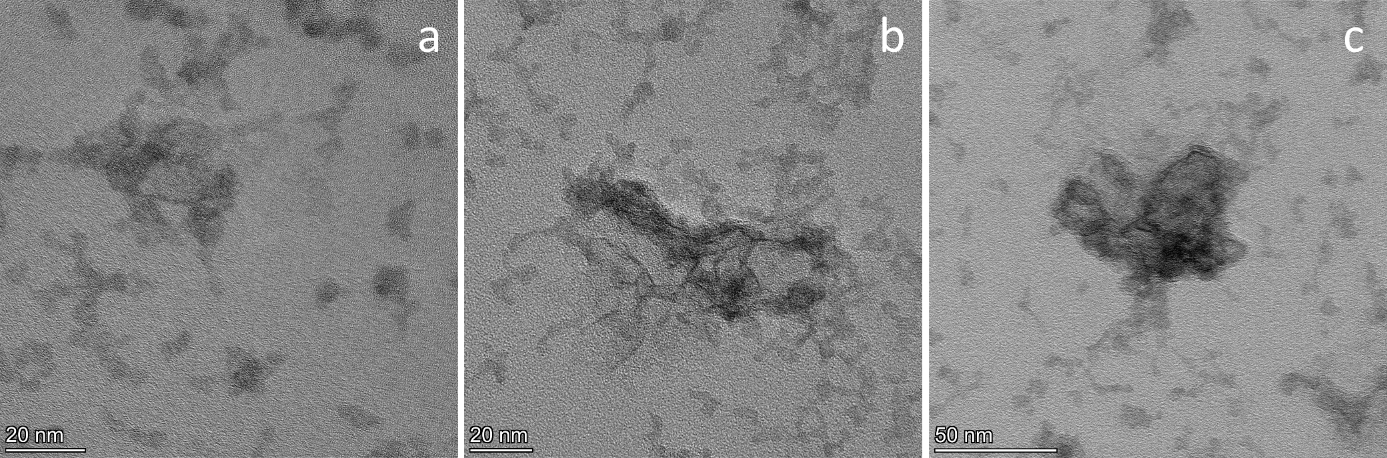


**Figure S4**. TEM images of the NPs prepared by Gd/Fe dual anodic dissolution in distilled water


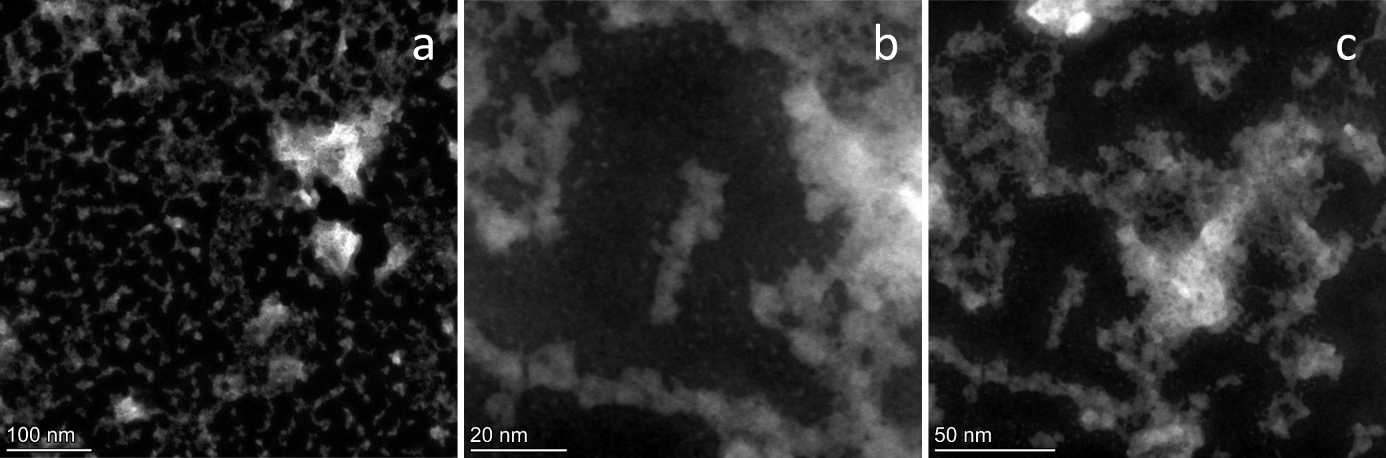


**Figure S5**. HAADF-STEM images of the NPs prepared by Gd/Fe dual anodic dissolution in distilled water

**SI-C. Further Raman measurements of the NPs**


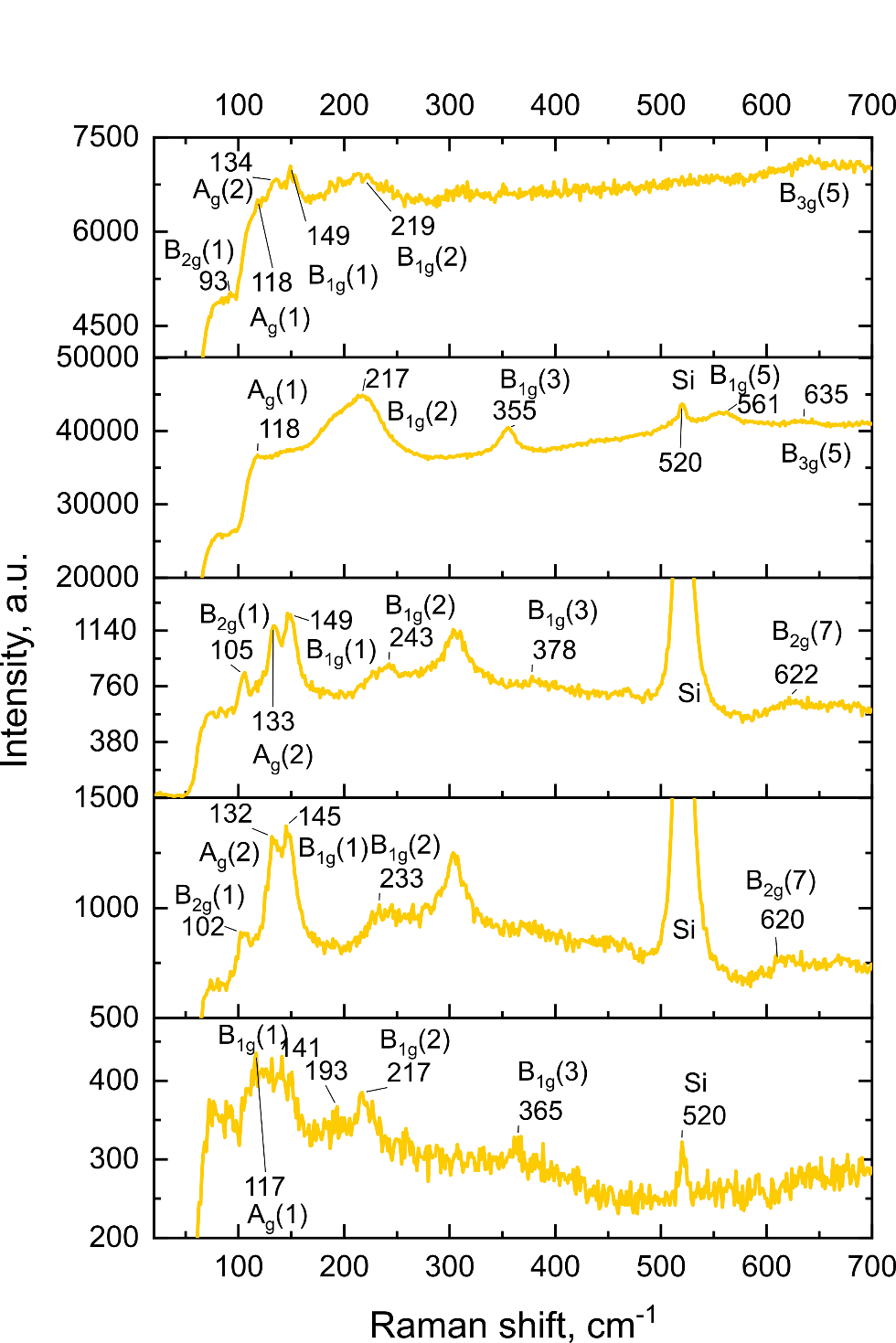


**Figure S6**. Raman spectra of the sample prepared by dual anodic dissolution of Gd/Fe in distilled H_2_O, the NPs were deposited on Si plate from colloid

**SI-D. Further FTIR measurements of the NPs**


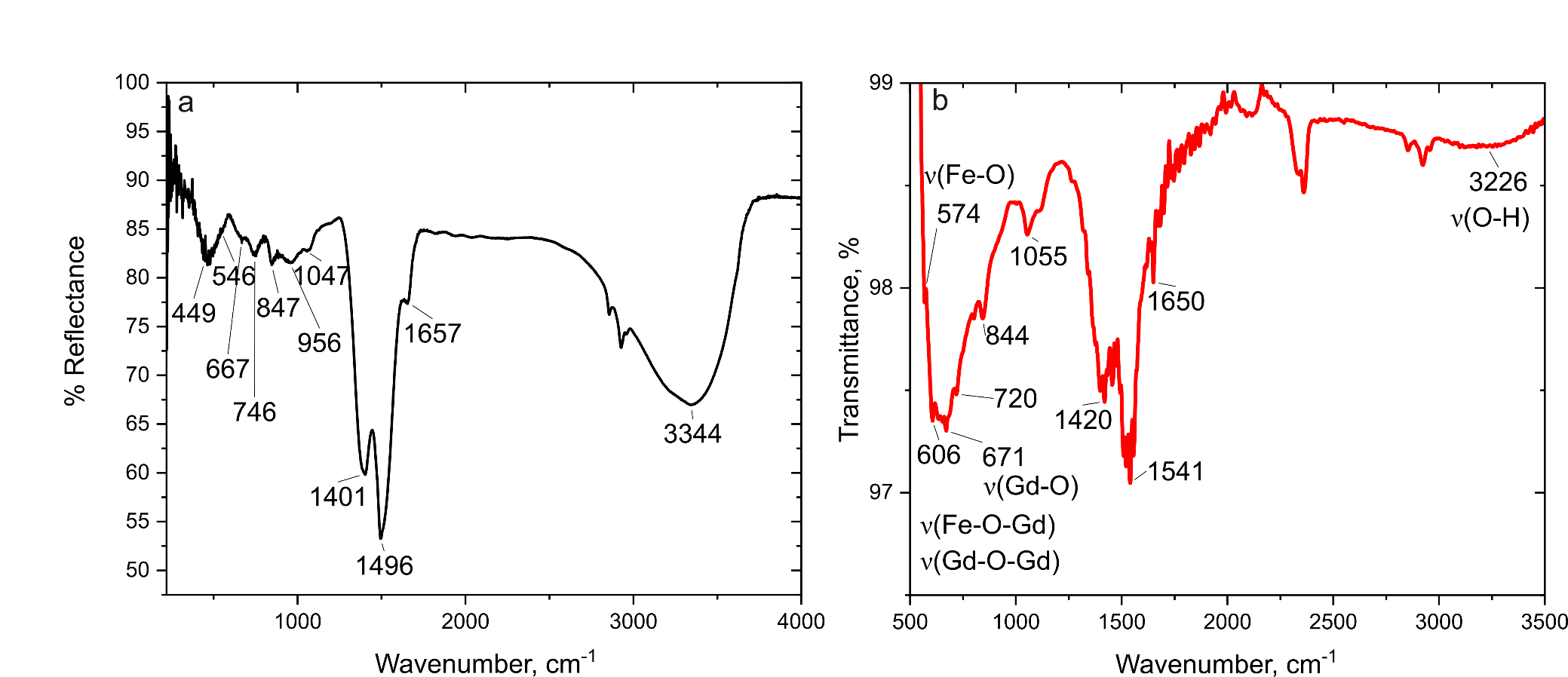


**Figure S7**. FTIR spectra of the GdFeO_3_ NPs prepared by Gd/Fe dual anodic dissolution in distilled H_2_O. The colloids were drop-casted onto Al substrate and dried at 80°C on a hot plate

**SI-E. Further XPS of the NPs**

**
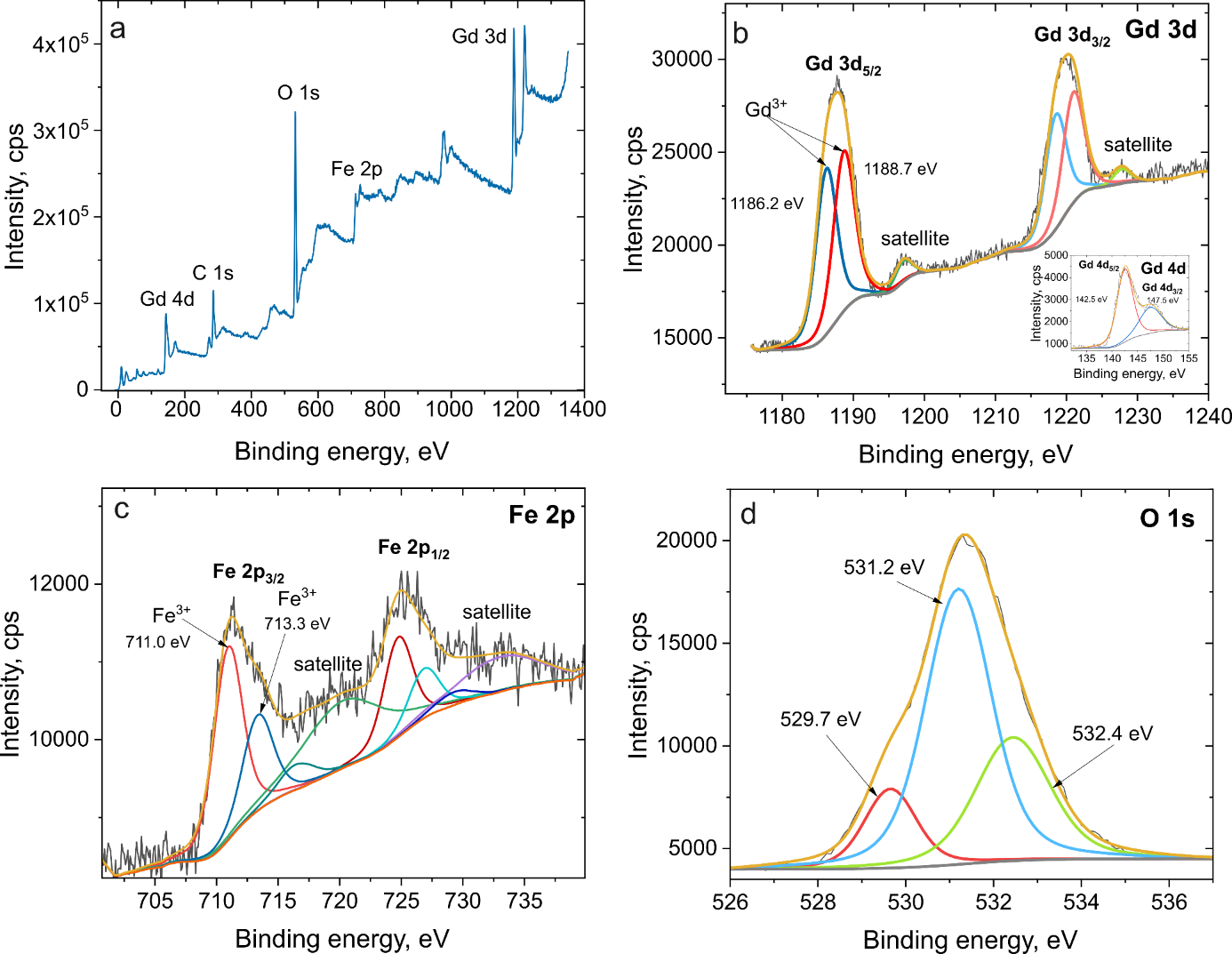
**

**Figure S8**. XPS spectra of the GdFeO_3_ sample prepared by dual anodic dissolution of Gd/Fe in distilled H_2_O: (a) survey spectrum, (b-d) – high-resolution deconvoluted core-level spectra for the Gd 3d (b), Fe 2p (c) and O 1s (d) levels; the inset in Figure S8b shows the high resolution spectra in the Gd 4d region


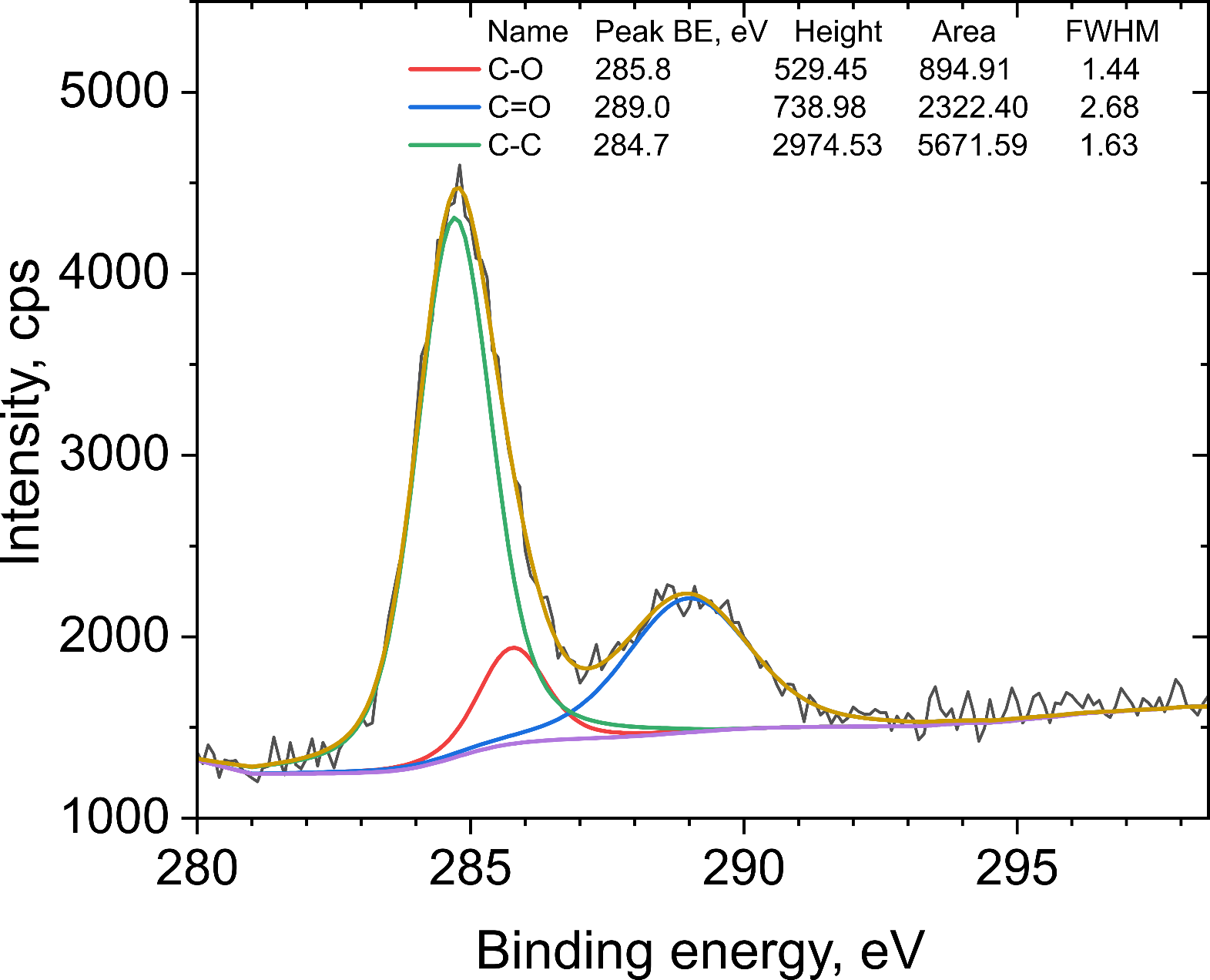


**Figure S9**. High-resolution deconvoluted core-level spectra of the GdFeO_3_ sample prepared by dual anodic dissolution of Gd/Fe in distilled H_2_O in the C 1s region

**SI-F. Further NP thin film characterization**


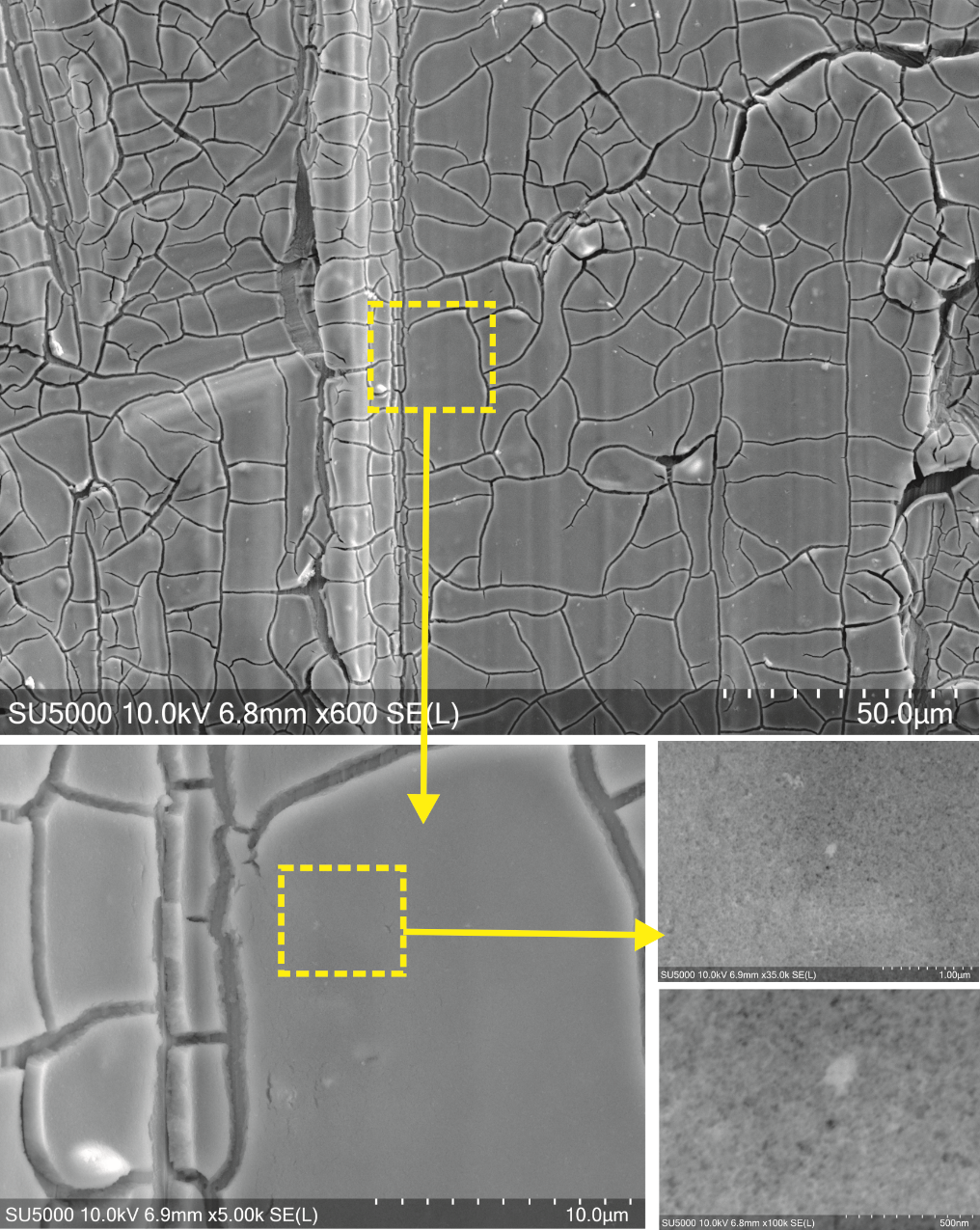


**Figure S10**. SEM images with different magnifications of the GdFeO_3_ NPs deposited onto Cu substrate during synthesis


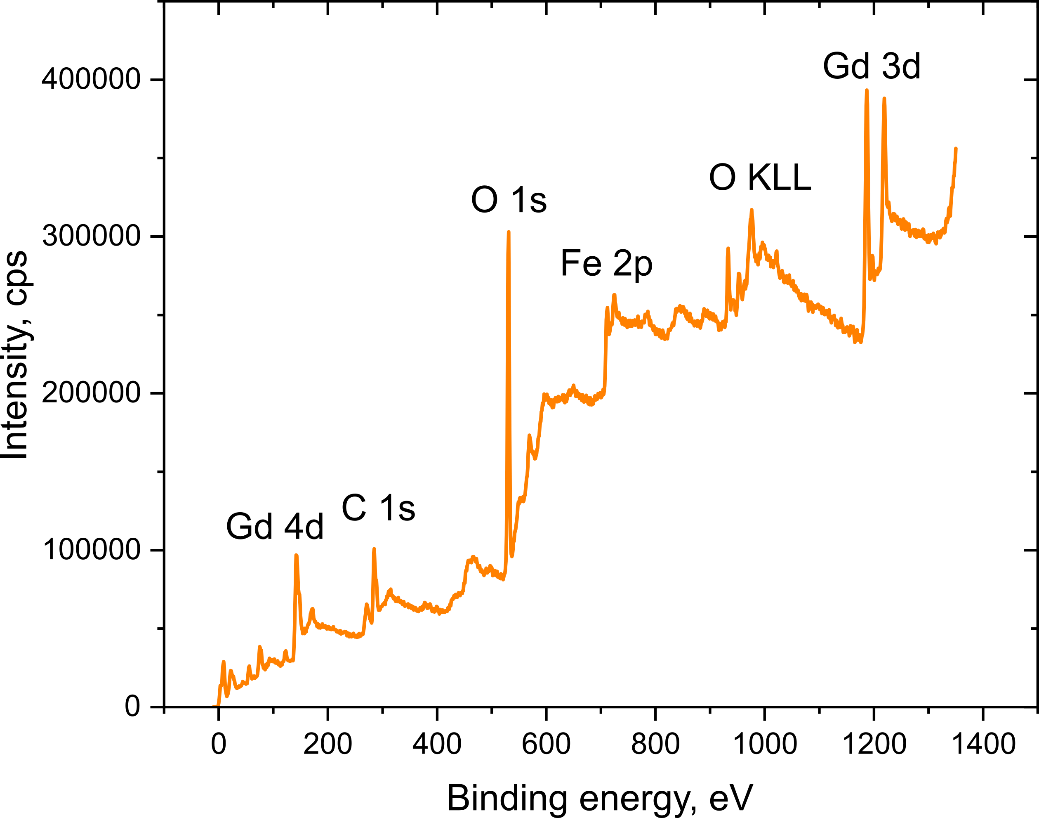


**Figure S11**. XPS survey spectrum of the GdFeO_3_ sample deposited on Cu during synthesis


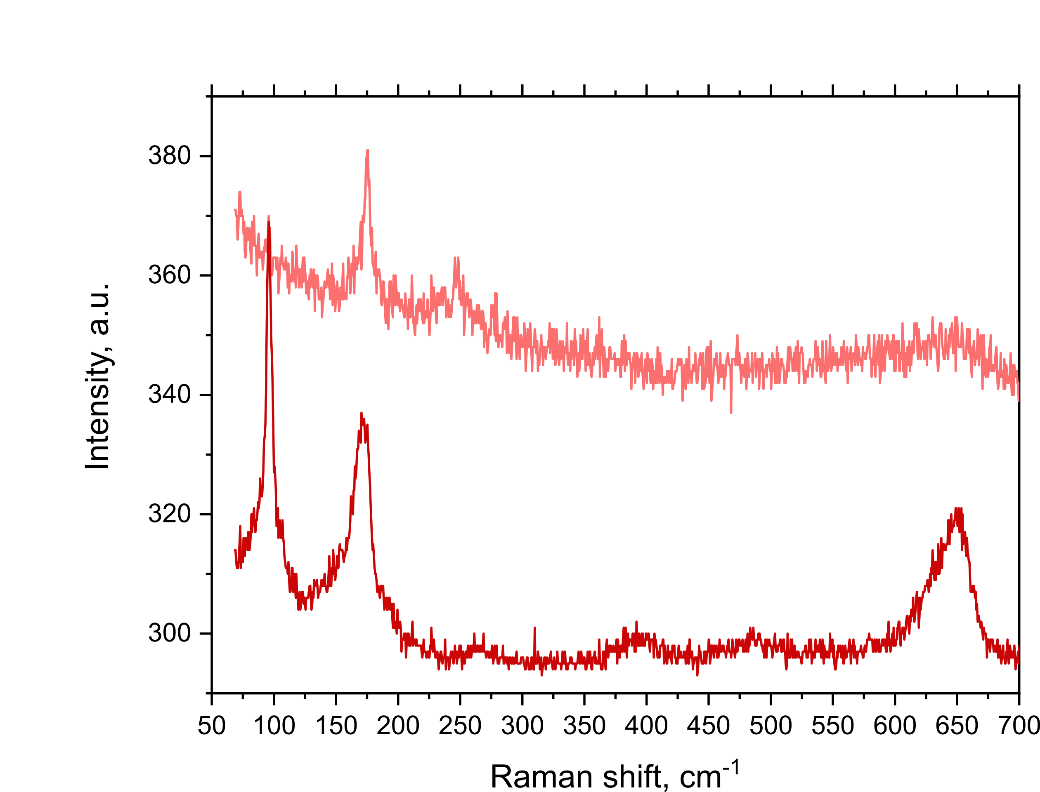


**Figure S12**. Raman spectra of the GdFeO_3_ sample prepared by Gd/Fe dual anodic dissolution with simultaneous deposition onto Cu substrate during synthesis

**SI-G. Details about NP size ranges**

The grain size can be determined from XRD results using Scherrer equation *D* = *Kλ* / *β*cos*θ*, where *D* is the nanoparticles crystallite size, *K* represents the Scherrer constant (0.94), *λ* denotes the X-ray wavelength (0.154 nm for Cu Kα), *θ* is the diffraction angle, *β* denotes the full width at half maximum (FWHM). The results of the calculations are presented in the table here below:

| peak | lambda, nm | K | 2θ, degree | Cosθ | beta | | Instr. broad., rad. | D, nm |
| --- | --- | --- | --- | --- | --- | --- | --- | --- |
|  |  |  |  |  | degree | rad |  |  |
| 110 | 0,154 | 0,94 | 22,99 | 0.201 | 1,360 | 0,0175 | 0,08 | 6,2 |
| 112 | 0,154 | 0,94 | 32,07 | 0.280 | 1,233 | 0,0215 | 0,08 | 7,5 |
| 021 | 0.154 | 0.94 | 35.12 | 0.304 | 1.308 | 0.0228 | 0.08 | 7.1 |

Due to the particles being closely interconnected, it is not possible to carry out a reliable statistical analysis based on TEM images. We include here a few more TEM images (figure S13) which report NPs with sizes ranging from ~5nm to 8 nm, values that are consistent with the XRD results.


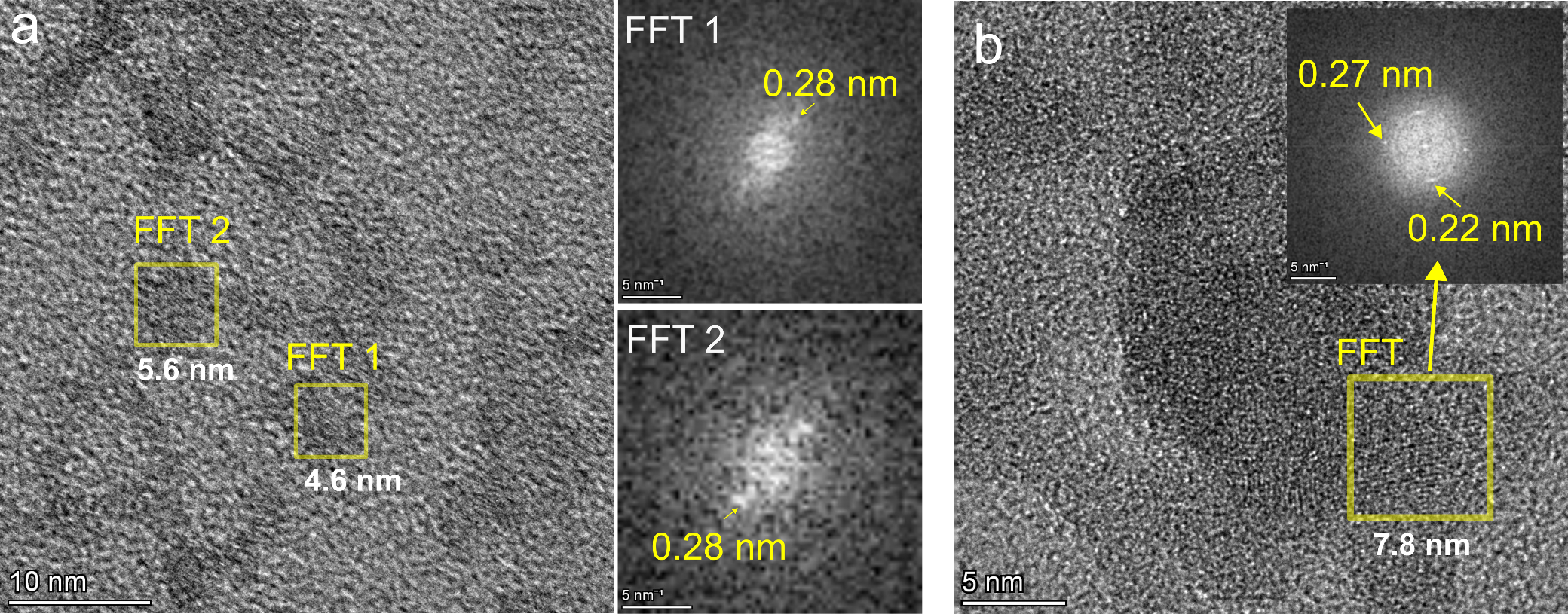


**Figure S13**. TEM images showing the approximated size of nanoparticles within the branched structures.
